# SPECT Perfusion Defects and Impaired Strain in Mild COVID-19: A Multimodal Imaging Study with a Female-Predominant Cohort

**DOI:** 10.3390/healthcare13050548

**Published:** 2025-03-04

**Authors:** Ji-Cheng Hsieh, Tanzim Bhuiya, Jonathan H. Sussman, Tony Dong, Danni Fu, David W. Wu, John Makaryus

**Affiliations:** 1 Department of Internal Medicine, Zucker School of Medicine at Hofstra/Northwell, New Hyde Park, NY 11549, USA; 2 Department of Internal Medicine, Hospital of the University of Pennsylvania, Philadelphia, PA 19104, USA; 3 Northwell Cardiovascular Institute, New Hyde Park, NY 11040, USA

**Keywords:** COVID-19, myocardial perfusion imaging, single-photon emission computed tomography, strain echocardiography, global longitudinal strain

## Abstract

**Background/Objectives:** This study investigates the association between mild first-wave COVID-19 infection and subclinical abnormalities in echocardiographic strain parameters and myocardial perfusion using single-photon emission computed tomography (SPECT). **Methods:** We conducted a retrospective analysis of patients from June 2020 to March 2021 with a history of mild first-wave COVID-19 infection, presenting with nonspecific cardiac symptoms and referred for SPECT myocardial perfusion stress testing. Patients had no obstructive coronary artery disease (CAD) on follow-up invasive angiography or cardiac computed tomography angiography (CCTA) and had transthoracic echocardiographic images of sufficient quality for strain analysis using AutoSTRAIN (TOMTEC^®^). **Results:** Fifteen patients met the inclusion criteria. SPECT and echocardiography were reviewed for perfusion and strain defects, respectively, in the inferior, anterior, lateral, and septal myocardial segments. All patients had at least one perfusion abnormality on SPECT: 2/15 (13%) had a fixed defect in one segment, 3/15 (20%) in two, 3/15 (20%) in three, and 7/15 (47%) in four. While 13/15 (87%) patients had normal qualitative findings on traditional echocardiography, 12/15 (80%) had abnormal global longitudinal strain (GLS) (>−18%) and transregional wall strain abnormality in at least one segment. Abnormalities on SPECT and strain echocardiography demonstrated a moderate but significant 60% concordance, with an intraclass correlation coefficient (ICC) of 0.486 (*p* = 0.028). **Conclusions:** Patients with ‘mild’ COVID-19 infection demonstrated a high frequency of abnormalities on SPECT myocardial perfusion imaging (even in the absence of obstructive CAD) which appeared to be concordant with abnormal strain parameters on echocardiography, suggesting possible subclinical effects on myocardial tissue.

## 1. Introduction

Severe SARS-CoV-2, or COVID-19, infection can lead to pro-coagulant and inflammatory dysregulation, most notably in the vascular endothelium. Cardiac involvement in COVID-19 patients can manifest broadly: myocardial injury, myocarditis, arrhythmias, acute coronary syndromes, heart failure, and microvascular dysfunction [1]. Although cardiac complications in severe COVID-19 cases are well-documented, the effects of mild first-wave infections—those not requiring hospitalization or showing biomarker leaks—remain less understood. Exercise intolerance, tachycardia, and chest pain are frequent long-term sequalae of COVID-19 infection, even in non-hospitalized individuals with mild infection, suggesting possible subclinical cardiovascular involvement [2,3].

Patients with prior COVID-19 infection have demonstrated abnormalities on cardiac imaging including cardiac MRI (cMRI), single-photo emission computed tomography myocardial perfusion imaging (SPECT), and strain echocardiography. cMRI findings in COVID-19 patients include myocardial edema, fibrosis, impaired right ventricular function, and inducible ischemia on stress perfusion cMRI [4]. This correlates with abnormal perfusion findings on SPECT in patients with prior COVID-19 infection reported in the literature, which has frequently led to further invasive evaluation [5,6]. Abnormalities of cardiac function on cMRI correspond with reported findings of abnormal global longitudinal strain (GLS) in patients with prior mild COVID-19 infection [7,8].

There are reports in the literature of abnormal perfusion on SPECT and abnormal cardiac function on cMRI and strain echocardiography in patients with mild COVID-19 infection, suggesting subclinical perfusion defects and subtle myocardial dysfunction and scarring. Our study aims to evaluate whether patients with a history of mild ‘first-wave’ COVID-19 and no prior obstructive coronary artery disease (CAD), as demonstrated on invasive coronary angiography or cardiac computed tomography angiography (CCTA), exhibit abnormal myocardial perfusion on SPECT and impaired myocardial function on echocardiographic strain analysis. We seek to isolate the potential effects of COVID-19 on myocardial perfusion and function and determine whether perfusion abnormalities correlate anatomically with functional regional wall strain parameters on echocardiography. Understanding these subclinical alterations could have significant implications for long-term cardiac monitoring and management of patients with a history of even mild COVID-19 infection, particularly during the first year of the global pandemic.

## 2. Materials and Methods

### 2.1. Study Design

Our single center retrospective cohort study screened patients between June 2020 and March 2021 who had recovered from ‘mild’ first-wave COVID-19 infection and were referred for SPECT myocardial perfusion imaging for cardiac complaints of chest pain or shortness of breath. Recovery was defined as the resolution of symptoms or negative subsequent COVID-19 PCR. ‘Mild’ infection was defined as not requiring hospitalization for supplemental oxygen or severe symptoms. Patients were included if they were over the age of 18 and had transthoracic echocardiographic images of sufficient quality for strain analysis after COVID-19 infection. Patients were excluded if they had active COVID-19 infection or elevated troponin at the time of SPECT imaging or if they had any history of myocarditis, myocardial infarction, or obstructive coronary artery disease, which may have been documented with either invasive coronary angiography (ICA) or coronary computed tomographic angiography (CCTA). Additionally, patients with a history of valvular heart disease, specifically severe mitral or aortic valvular disease, were excluded to minimize confounding effects on global longitudinal strain measurements. Collected data included demographic information, comorbidities, and the results of SPECT imaging, echocardiographic imaging, and ischemic evaluation with ICA or CCTA. SPECT imaging was performed without computed tomography-based (CT) attenuation correction.

### 2.2. Data Collection

Screening of the COVID-19 patient cohort was completed with nuclear and echocardiography reporting software (CardioReportWare, CRW, http://www.cardioreportware.com/). Demographic and comorbidity data, as well as the results of SPECT and echocardiography imaging, were obtained via review of the electronic health record by multiple reviewers. Strain analysis was conducted using AutoSTRAIN (TOMTEC^®^). Echocardiographic images were reviewed with one attending cardiologist with expertise in advanced echocardiographic imaging. As this retrospective study utilized de-identified patient data, informed consent was not collected from the patients included in the study.

### 2.3. Statistical Analysis

Statistical analysis was performed using Python v3.11 and R v4.4.0. SPECT myocardial perfusion abnormalities by anatomic segments were binarized to facilitate comparison with wall motion abnormalities (WMA) on strain echocardiography. Abnormalities were organized into an assessment of only four anatomic segments (inferior, anterior, lateral, and septal) to simplify comparison between SPECT and strain echocardiography. The four anatomic segments were collectively evaluated using the intraclass correlation coefficient (ICC) with the Pingouin package in Python, specifically ICC3. Categorical variables were assessed using chi-squared tests, while quantitative variables were compared using paired or unpaired two-sided t-tests as appropriate; *p*-values <0.05 were considered statistically significant.

## 3. Results

As these data was acquired from patients during the first year of the pandemic, during which the volume of stress testing steeply declined, only fifteen patients met the inclusion criteria and were included in the study cohort. Age, demographic, and comorbidity information is summarized in Table 1. The mean age was 64 years (a SD of 11.07 with a range of 45–87 years). A total of 14/15 (93%) patients were female. Of the patient cohort, 8/15 (53%) had a history of hyperlipidemia, 4/15 (26%) had a history of diabetes mellitus, 9/15 (60%) had a history of hypertension, 2/15 (13%) had a history of smoking, and 8/15 (53%) had a history of obesity. On ICA or CCTA, 13/15 (87%) patients had no flow-limiting lesions seen and 2/15 (13%) patients had non-obstructive coronary artery disease defined as less than 50% stenosis.

SPECT imaging revealed that all patients exhibited at least one mild to moderate perfusion abnormality in one of the four major anatomic regions (inferior, anterior, lateral, and septal). A total of 2/15 (13%) patients demonstrated a fixed perfusion defect in one segment, 3/15 (20%) in two, 3/15 (20%) in three, and 7/15 (47%) in four. Defects were evenly distributed by anatomic region: 11/15 (73%) patients had an inferior defect, 13/15 (87%) had an anterior defect, 11/15 (73%) had a lateral defect, and 10/15 (67%) had a septal defect. On echocardiographic imaging, 13/15 (87%) patients had completely normal left ventricular function, and 2/15 (13%) patients had mild to moderate left ventricular systolic dysfunction. A total of 14/15 (93%) patients had no valvular abnormalities seen, and only 1/15 (7%) patient demonstrated mild–moderate mitral regurgitation and moderate–severe tricuspid regurgitation on echocardiographic imaging. A total of 12/15 (80%) patients demonstrated an abnormal GLS, defined as >−18%, with an average GLS of −16.78 (SD 4.2, range −6.7 to −25.4). A total of 12/15 (80%) patients had abnormal strain in at least one anatomic area: 1/15 (7%) in one, 3/15 (20%) in two, 4/15 (27%) in three, and 4/15 (27%) in four. Strain abnormalities by anatomic area noted 8/15 (53%) patients having an inferior abnormality, 8/15 (53%) having an anterior abnormality, 7/15 (47%) having a lateral abnormality, and 12/15 (80%) having a septal abnormality. SPECT and echocardiographic imaging results are summarized in Table 2 and Table 3, respectively.

A comparison of the SPECT and echocardiographic imaging results demonstrated a 60% concordance of anatomic distribution of perfusion defects on SPECT and strain defects on echocardiography. The intraclass correlation coefficient (ICC) was 0.486 (*p* = 0.028), indicating a statistically significant level of agreement between the two imaging methods. While the ICC value (0.486, *p* = 0.028) suggests moderate agreement, the overall concordance rate between perfusion defects and strain abnormalities was 60% (36/60 segments), supporting the relevance of these findings. The concordance analysis is further described in Table 4. Furthermore, analysis with a two-proportion z-test found that the degree of concordance—whether patients had low (0, 1, or 2 matching measurements) or high (3 or 4 matching measurements) concordance—was not significantly affected by any of the comorbidities assessed (*p* > 0.05).

## 4. Discussion

We evaluated a cohort of patients who recovered from mild first-wave COVID-19 and were later referred for SPECT myocardial perfusion imaging due to cardiac complaints at the peak of the pandemic. We found that, although the patients in this cohort had no obstructive coronary artery disease on CCTA or ICA, all of the patients demonstrated abnormal perfusion on SPECT with at least one fixed defect in an anatomic distribution. Additionally, the majority of patients demonstrated abnormal GLS with at least one strain abnormality in an anatomic distribution. We found that the anatomic distribution of fixed defects on SPECT were significantly concordant with the anatomic distribution of strain defects on echocardiography, and analysis of concordance stratified by comorbidities measured did not demonstrate any effect of comorbidities such as hyperlipidemia, diabetes mellitus, smoking history, or hypertension on the degree of concordance. The ICC of 0.486 suggests moderate agreement; however, this should be interpreted in conjunction with the 60% accuracy rate observed in the segmental analysis.

COVID-19’s mechanism of cardiac injury is suspected to be due to microvascular changes, as Bilge et al. found that 24% of patients with prior COVID-19 pneumonia showed ischemia on SPECT-MPI, with pneumonia identified as an independent predictor of ischemia [5]. Araz et al. reported a significant rise in ischemia on SPECT imaging in patients with recent COVID-19 requiring further coronary angiography and stenting [6]. We also observed this phenomenon, where patients with prior COVID-19 infection presented to our cardiology clinic with cardiac chest pain and shortness of breath, were found to have abnormal SPECT imaging, and were subsequently referred for ICA. Abnormal longitudinal strain (GLS) on echocardiography has also been observed in patients with mild COVID-19, suggesting subclinical myocardial dysfunction. Gherbesi et al. reported that left ventricular GLS was notably reduced in young adults who had recovered from mild COVID-19 compared to healthy controls (−22.7 ± 1.6% vs. −25.7 ± 2.3%; *p* < 0.01) [7]. Rácz et al. found that GLS values remained lower in patients two months after mild COVID-19 infection compared to controls (−19.1% vs. −20.3%; *p* < 0.01) [8].

Our findings suggest that patients with prior history of ‘mild’ first-wave COVID-19 infection frequently present with subtle perfusion abnormalities on SPECT suggestive of myocardial scar from ongoing microvascular insult, along with abnormal strain echocardiography. This is consistent with perfusion cMRI findings in ‘severe’ COVID-19 suggesting occult coronary artery disease and myocardial scar [9]. In our study, we found that these abnormalities were otherwise not detectable on formal ischemic evaluation or traditional echocardiography. In sum, in light of COVID-19’s known prothrombotic effects, infection by both ‘mild’ and ‘severe’ COVID-19 may lead to microvascular injury and scarring that is recognized on multimodal imaging.

We noted that our cohort of symptomatic patients demonstrated abnormalities on SPECT imaging and strain echocardiography. SPECT imaging was performed without CT attenuation correction, and our described perfusion defects could represent attenuation artifacts. Attenuation correction, however, has been shown to have a non-significant benefit on diagnostic accuracy, and it would be difficult to explain the significant concordance between the results of SPECT and strain imaging to simply attenuation artifact alone [10]. It is unclear if asymptomatic patients with prior COVID-19 also demonstrate these abnormal findings. It is unclear whether mild COVID-19 infection predisposes symptomatic patients with abnormal SPECT and echocardiographic findings to developing overt ischemic heart disease or heart failure, detectable through ICA or CCTA. Regardless of these limitations, our study adds to the existing literature supporting COVID-19’s prothrombotic effects. Our study also presents novel data specifically correlating SPECT imaging findings with strain echocardiography, identifying a notable concordance between abnormal imaging findings in patients with prior ‘first-wave’ COVID-19 infection that warrants further investigation.

## 5. Limitations

Our study is limited by a small sample size from a single academic center. Our notable preliminary data warrants future expansion of the cohort to multiple sites. Additionally, given the substantial differences between early and present-day COVID-19 strains, caution should be exercised when applying these findings to current clinical populations. The inclusion of patients with alternative strains of COVID-19 beyond ‘first-wave’ COVID-19 would also improve generalizability.

Our study cohort was predominantly female (93%), which limits the generalizability of our findings to male patients. Future studies should aim for a more balanced sex distribution to assess potential sex-specific differences in myocardial involvement post-COVID-19. Additionally, a significant proportion (53%) of the study cohort was obese, which may lead to inferior wall soft-tissue attenuation artifacts on SPECT [11]. Future studies warrant the addition of attenuation correction to maximize diagnostic accuracy.

Another notable limitation is the absence of a control group. This study was designed to characterize abnormal imaging findings exclusively in patients with prior COVID-19 infection. Subsequent studies would benefit from comparisons to age- and comorbidity-matched control populations to better isolate the impact of COVID-19 on myocardial function. While our findings align with prior studies using similar imaging modalities, the small sample size and lack of a control group limit definitive conclusions about causality.

Our study highlights ‘first-wave’ COVID-19 survivors as a population of interest that may be predisposed to myocardial scarring, and we provide preliminary evidence warranting further investigation in larger, controlled studies. Longitudinal studies, including repeat SPECT imaging, are needed to determine whether the observed perfusion abnormalities persist and correlate with adverse cardiovascular outcomes.

## 6. Conclusions

This study provides preliminary evidence that symptomatic patients recovering from mild COVID-19 exhibit myocardial perfusion defects on SPECT and abnormal global longitudinal strain on strain echocardiography, suggesting potential subclinical cardiac involvement. These findings highlight the need for further research using larger, controlled studies to assess the clinical significance and persistence of these abnormalities.

## Figures and Tables

**Table 1:** Study cohort demographics and comorbidities.

Demographics and Comorbidities	Values
Age (Years)	Mean 64 (SD 11.07, Range 45–87)
Female Sex (Count, %)	14 (93%)
Hyperlipidemia (Count, %)	8 (53%)
Diabetes Mellitus (Count, %)	4 (26%)
Hypertension (Count, %)	9 (60%)
Smoking History (Count, %)	2 (13%)
Obesity	8 (53%)

**Table 2:** SPECT myocardial perfusion imaging results, with defects by anatomic distribution (Mild = 1, moderate = 2, and severe = 3).

Patient Number	Perfusion Defect (Inferior)	Perfusion Defect (Anterior)	Perfusion Defect (Lateral)	Perfusion Defect (Septal)
1	0	0	1	0
2	2	1	2	2
3	0	2	2	0
4	2	2	2	1
5	2	0	0	2
6	2	2	0	0
7	1	1	1	2
8	2	2	2	2
9	2	1	0	2
10	2	2	1	2
11	2	2	2	2
12	1	1	1	1
13	0	2	0	0
14	1	1	1	0
15	0	1	1	2

**Table 3:** Strain echocardiography imaging results, with measurements of global longitudinal strain (GLS) and strain abnormalities by anatomic distribution (Present = 1; absent = 0).

Patient Number	Strain Abnormality (Inferior)	Strain Abnormality (Anterior)	Strain Abnoramlity (Lateral)	Strain Abnormality (Septal)	Global Longitudinal Strain (GLS) Value (%)
1	1	0	0	1	−16.80
2	1	1	1	1	−13.50
3	0	0	0	1	−16.50
4	1	1	1	1	−12.80
5	0	0	0	0	−21.30
6	0	0	0	0	−17.50
7	1	1	0	1	−17.20
8	0	0	1	1	−20.30
9	1	1	0	1	−14.30
10	1	1	1	1	−16.90
11	1	1	1	1	−6.70
12	0	0	0	0	−25.40
13	0	0	1	1	−18.80
14	0	1	1	1	−17.60
15	1	1	0	1	−16.10

**Table 4:** Matching analysis of SPECT and echocardiographic imaging, with degree of concordance analysis by anatomic distribution. (Low concordance = 0–1; high concordance = 3–4.)

Patient Number	Matched Findings (Inferior)	Matched Findings (Anterior)	Matched Findings (Lateral)	Matched Findings (Septal)	Degree of Concordance (0–4)	Strength of Concordance (Low–High)
1	False	True	False	False	1	Low
2	True	True	True	True	4	High
3	True	False	False	False	1	Low
4	True	True	True	True	4	High
5	False	True	True	False	2	Low
6	False	False	True	True	2	Low
7	True	True	False	True	3	High
8	False	False	True	True	2	Low
9	True	True	True	True	4	High
10	True	True	True	True	4	High
11	True	True	True	True	4	High
12	False	False	False	False	0	Low
13	True	False	False	False	1	Low
14	False	True	True	False	2	Low
15	False	True	False	True	2	Low

## Data Availability

Data are contained within the article.
